# Lipase from new isolate Bacillus cereus ATA179: optimization of production conditions, partial purification, characterization and its potential in the detergent industry

**DOI:** 10.3906/biy-2101-22

**Published:** 2021-06-23

**Authors:** Elif DEMİRKAN, Aynur AYBEY ÇETİNKAYA, Maoulida ABDOU

**Affiliations:** 1 Department of Biology, Faculty of Arts and Sciences, Bursa Uludağ University, Bursa Turkey

**Keywords:** Contaminated fabric, enzyme, enzymatic properties, screening, tributyrin assay

## Abstract

In this study, 341 *Bacillus* sp. strains were isolated from agricultural soils of Turkey. The potent extracellular lipase producer was selected. It was identified by 16S rRNA, named as *Bacillus cereus* ATA179. Optimal nutritional and physical parameters for lipase production were determined. Sucrose as the carbon source, (NH_4_)_2_HPO_4 _as the nitrogen source, CaCl_2_ as the metal ion were obtained. The best results of physical parameters were stated at 45°C, pH 7.0, shaking rate 50 rpm, inoculation amount 7% and inoculum age 24 h. ATA179 strain showed a 51% increase in enzyme production in the modified medium created by optimizing nutritional and physical conditions. Optimum pH value and temperature were found as 6.0 and 55 °C, respectively. CaCl_2_, Tween 20, Triton X-100 had an activating effect on enzyme activity. V_max_ and K_m _kinetic values were found as 18.28 U/mL and 0.11 mM, respectively. The molecular weight was determined as 47 kDa. Lipase was found to be stable up to 75 days at -20 ºC. The potential of the enzyme in detergent industry was also investigated. It was not affected by detergent additives, but was found to be effective in removing oils from contaminated fabrics. This new lipase may have potential to be used in detergent industry.

## 1. Introduction

Enzymes, which have important metabolic functions for cellular structures, have entered daily and economic life to be used for various purposes. Industrial use of enzymes has become widespread since 1960s (Aehle, 2004). The most important enzymes used in the industry are amylases, proteases, lipases, and phytases. Lipases are enzymes that break down the glycerol esters of fatty acids (Babu and Rao, 2007). They are a physiologically and commercially important group of enzymes as their use increases rapidly and steadily for various biotechnological applications (Jaeger and Eggert, 2002). The uses of microbial lipase market is estimated to be USD 425.0 million in 2018 and it is projected to reach USD 590.2 million by 2023, growing at a CAGR of 6.8% from 2018 (Chandra et.al., 2020). In general, lipases have promising applications in detergent formulation, organic chemical processing, agrochemical industry, biosulfonate synthesis, food, pharmacy, cosmetics, and paper manufacturing (Houde et al., 2004). Lipases are used in both dishwashing and laundry formulations commercial detergents for the removal of lipid stain, sebum, and fatty food stains from fabrics. Approximately 32% of the microbial lipases produced in the world are used in the detergent industry as it forms the very important compound in detergents (Barros et al., 2010). The use of lipases in the detergent industries is increasing day by day.

Approximately 96% of enzymes used for industrial purposes are produced from microorganisms (Wolfgang, 2004). In the industry, mostly bacteria and fungal lipases are preferred. Bacterial lipases were first observed in the strains *Pseudomonas auroginosa* and *Serratia marescens* species in the year 1901 (Eijkmann, 1901). *Achromobacter, Alcaligenes, Arthrobacter, Bacillus, Burkholderia, Chromobacterium,* *Geobacillus,* and *Pseudomonas* genera are important sources of bacterial lipase (Sarmah et al., 2018). *Bacillus* species have an important place among bacteria. Some *Bacillus* species have thermostable lipase production. Thermostable lipases are preferred in the industry (Bhosale et al., 2016). 

The first stage of enzyme production by microbial route is the selection of microorganism at nature. Culture media and fermentation conditions are also important parameters affecting enzyme production. In addition, it is important to reveal the properties of enzymes obtained from new isolates by purification (Gupta et al., 2004).

In this study, the screening of lipase enzyme production capacities from previously isolated 341 *Bacillus* strains, optimization of growth conditions, characterization by partial purification, and its potential in the detergent industry were investigated.

## 2. Materials and methods

### 2.1. Materials 

White fabrics (50% polyester (PES) + 50% cotton) were obtained from Bursa Uludag University, Department of Textile Engineering. The pollutants (olive oil and grease) and different branded detergents (3 solids and 3 liquids) used in the study were purchased from markets.

### 2.2. Qualitative screening of lipase positive bacteria

In this study, 341 *Bacillus* sp. strains that were obtained from our previous studies were used (Usta and Demirkan, 2013; Demirkan et al., 2014; Demirkan et al., 2020) The *Bacillus* sp. strains were screened for lipase production and cultivated on TBA (Tributyrin Agar) solid medium containing (g/L) meat extract 3, peptone 5, tributyrin 10 mL, and agar 20 (pH 7.0) at 37 ^o^C for 48 h (Kumar et al., 2012). After incubation, colonies forming clear zone on the petri dish were accepted as positive for lipase. The diameters of the colony and the hydrolysis zones around colonies were measured by ruler. The following formula was used for calculating the Enzymatic Index (EI) (Florencio et al., 2012).

EI = Diameter of hydrolysis zone (1)

 Diameter of colony

The strain showed the largest EI value was chosen and assays were continued with this strain.

### 2.3. Bacterial identification using 16S rRNA sequencing

Bacteria identification and phylogenetic analysis were carried out by REFGEN Biotechnology (Ankara, Turkey) company. *Bacillus* genomic DNA was extracted for the bacterial identification and phylogenetic analysis (Qbiogene, Montreal, PQ, Canada). The sequence analysis was performed using ABI 3100 Genetic Analyzer (Applied Biosystems, Waltham, MA, USA). The obtained sequences were compared with those desosited in the GenBank database (The National Center for Biotechnology Information-NCBI, Bethesda, MD, USA) using BLAST (Altschul et al., 1990). The 16S rRNA sequences of strain was aligned with other *Bacillus* species using CLUSTAL W program (Thompson et al., 1994). The phylogenetic analysis was done by MEGA 6.0 software, using the neighbor-joining method (Saitou and Nei, 1987). The sequence was submitted to genbank and accession number was obtained.

### 2.4. Quantitative assay of lipase

To determine the best enzyme production, three different media (Kumar et al., 2005; Hasan et al., 2006; Dahiya and Purkayastha, 2011) were used and compared with each other. Among the culture media, Kumar et al. (2005) medium exhibited a significant impact on enzyme production. This medium contained (w/v %) yeast extract 0.5, peptone 0.5, CaCl2 0.005, NaCl 0.05, olive oil 1 (pH 7.0). Overnight precultures adjusted at 1 × 108 CFU/mL were inoculated at 5% in medium and incubated at 37 °C for 72 h in 150 rpm. Bacterial growth and enzyme activity were performed at the 16th, 24th, 40th, 48th, 64th, and 72th hours. The optical density at 600 nm of bacterial growing was followed by a spectrophotometer (Beckman Coulter-UD 700).

### 2.5. Lipase activity assay 

Lipase activity was assessed by titrimetric analysis (Sugihara et al., 1991). Reaction mixture containing 4.5 mL of 50 mM Tris-HCl (pH 7.0), 0.5mL of 0.1 M CaCl_2, _1 mL of olive oil and 1 mL of crude enzyme solution were incubated in a water bath shaker (150 rpm) at 30 °C for 30 min. The enzymatic reaction was stopped by the adding 20 mL of 99.8% ethanol. The pH value of the incubation medium was titrated to 10.5 using a burette containing 50 mM KOH. One unit lipase activity was described as the amount of enzyme releasing 1 µmol of fatty acid under experimental conditions.

### 2.6. Optimization of the bacterial growth conditions for lipase

In this study, nutritional and physical parameters were optimized for production of lipase by *Bacillus* sp. Various carbon sources (1%) such as castor oil, coconut oil, corn oil, glucose, maltose, sucrose, starch, soybean oil, sunflower oil, and olive blackwater waste evaluated.

As organic nitrogen sources (1%) on enzyme production yeast extract, corn step liquor, peptone, tryptone, and (NH_4_)_2_HPO_4_, KNO_3_, (NH_4_)_2_NO_3_, (NH_4_)_2_SO_4_ as inorganic nitrogen sources were used. The effect of different metal ions (0.055%), such as MnSO_4_, FeSO_4_, LiSO_4_, BaCl_2_, KCl, NaCl, CaCl_2_, CuSO_4_,were studied for this purpose. For the physical optimization of the medium, different temperatures (35-60 °C), pH ranges (4.0-9.0), inoculum amounts (5%-10%), inoculum ages (18-48 days) and agitation (0-250 rpm) were investigated.

As a result of the experiments, a new medium was obtained by combining the best nutritional and physical factors. Enzyme production in this modified medium was compared with basal medium. 

### 2.7. Partial purification of lipase 

The crude lipase enzyme from *Bacillus* sp. was centrifuged at 5000 rpm for 15 min at 4 °C. The supernatant was precipitated with 20%-80% ammonium sulphate fractionation. The precipitates were collected (10,000 rpm, 30 min at 4 ^ο^C), dissolved in 50 mM Tris-HCl buffer (pH 7.0) containing 1 mM CaCl_2 _and then dialyzed overnight in the same buffer at 4 ^ο^C. After dialysis, samples were concentrated using ultrafiltration through a Centriprep-10 concentrator (Amicon). Lowry et al. (1951) method was used to determine the protein content.

### 2.8. Characterization of partially purified lipase enzyme

To determine the optimum temperature, enzyme was incubated between 30–80 °C. The optimum pH of enzyme was determined by using some buffers; 0.1 M glycine-HCl (pH 2.0 and 3.0), 0.1 M sodium acetate (pH 4.0-6.0), 0.1 M Tris-HCl (pH 7.0 and 8.0) and 0.1 M glycine-NaOH (pH 9.0 and 10). Temperature and pH value stability were also tested. To detect the effect of metal ions, salts and reducing compounds on enzyme activity, 1 and 5 mM FeSO_4_, MnSO_4_, MgSO_4, _ZnSO_4_, CuSO_4_, CaCl_2_, NaCl, LiSO_4_, BaCl_2,_ KCl, EDTA, SDS, Tween 20 and Triton X-100 Tween 20 were used. Relative activities (%) were calculated taking the untreated enzyme activity noted as 100%.

For kinetic analysis, tributyrin concentration was ranged from 0.1–1.2 mM and enzyme activity was assessed K_m_ and V_max_ were calculated from the Lineweaver-Burk plot. The molecular weight of the enzyme was estimated by SDS-PAGE(Laemmli, 1970). To determine the storage temperature of the crude lipase, enzyme was stored at room temperature, 4 °C and –20 °C upto 105 days, and the residual activity was calculated at each 15 days. 

### 2.9. Determination of potential use of the lipase enzyme in the detergent industy

To examine the effect of detergent additives on lyophilized lipase activity, the enzyme was incubated with 1 and 5% Triton X-100, EDTA, SDS and H_2_O_2_ at 55 and 65 ^o^C for 1 h at 100 rpm. The change in lipase activities was calculated as relative activity (%) based on the initial activities.

For lipase application, a white fabric containing 50% cotton and 50% polyester (PES), was used. Olive oil and grease oil were taken to trial as contaminants. Detergents available in the market (3 solids and 3 liquids) were selected from different brands to be tested. The experimental study was done as follows: contaminated fabric (control), contaminated fabric + 1 mL of lipase, contaminated fabric + 1 mL solid detergent, contaminated fabric + 1 mL solid detergent + 1 mL lipase, contaminated fabric + 1 mL liquid detergent, and contaminated fabric + 1 mL liquid detergent + 1 mL lipase. The fabric pieces were cut to a size of 60 *×* 60 mm, and contaminated separately with olive oil and grease oil to cover the fabric. The fabrics treated were incubated at 37 ºC for 1 h in 90 *×* 90 mm diameter petri dishes by applying the above conditions. After incubation, the fabrics were gently rubbed with a brush and rinsed with distilled water and dried. Contaminated fabrics were treated with lipase enzyme and detergents and whiteness indexes after enzyme, and detergent applications were measured using Konica Minolta CM3600-D color measurement spectrophotometer. Uncontaminated fabric was used to standardize the color measurement spectrophotometer. The “whiteness” indexes of the control and treated samples were compared. 

### 2.10. Statistical analysis

Statistical analysis of experimental results was performed using student’s t-test that was calculated using excel spread-sheets available in Microsoft Excel. Results are the means of three independent determinations and bars correspond to standard deviations.

## 3. Results

### 3.1. Screening and identification of lipase positive bacterial strain

**Figure 1:**
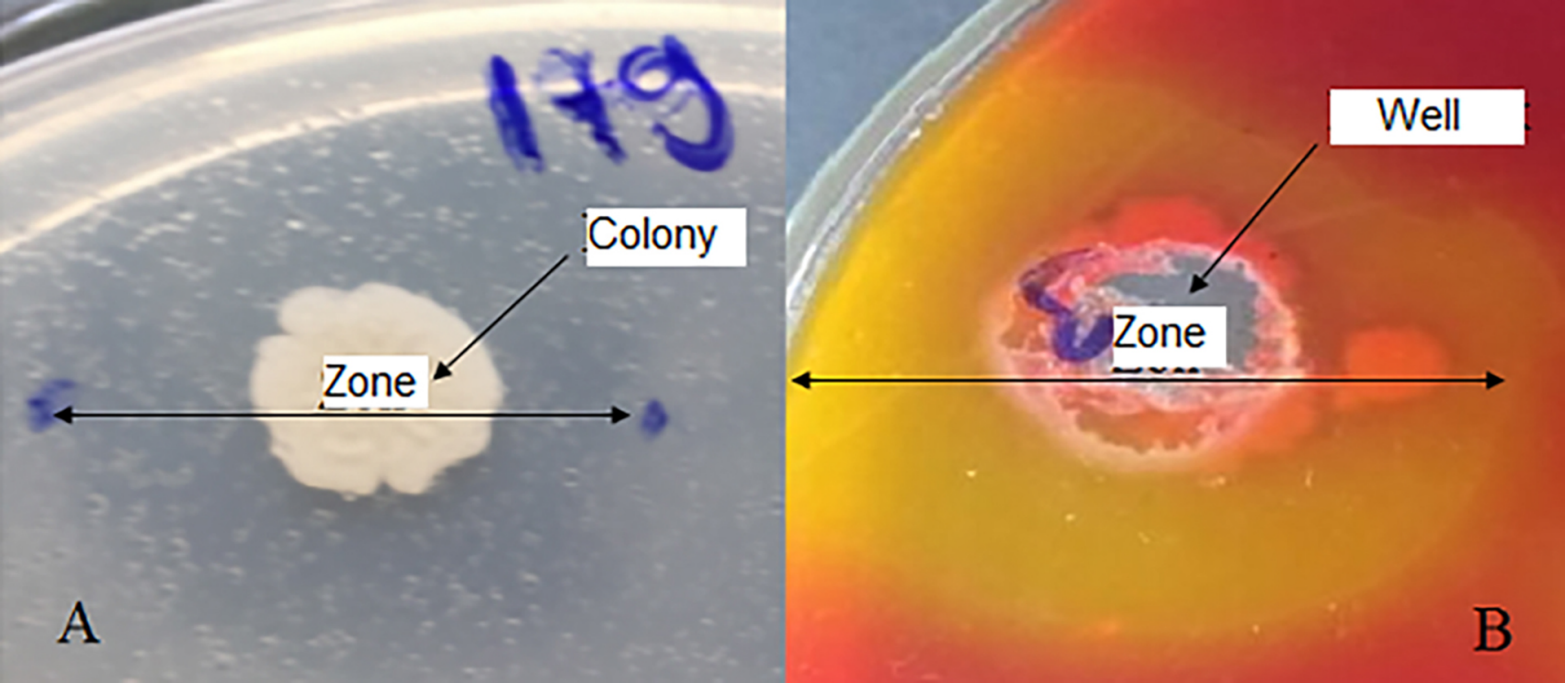
Clear zone by lipase-producing Bacillus sp. ATA179 on TBA (Tributyrin Agar) plate after 48 h (A) and hydrolytic zone on phenol red agar (B).

Three hundred and forty-one *Bacillus* strains used in the study, 141 of them were obtained as potential extracellular lipase producer. Of these 141 strains 74 displayed weak (EI = 0.07–0.30), 42 medium (EI = 0.30–0.50) and 25 large hydrolytic zones (EI = 0.5–2). One strain with EI = 2 was selected (Figure 1). 

**Figure 2:**
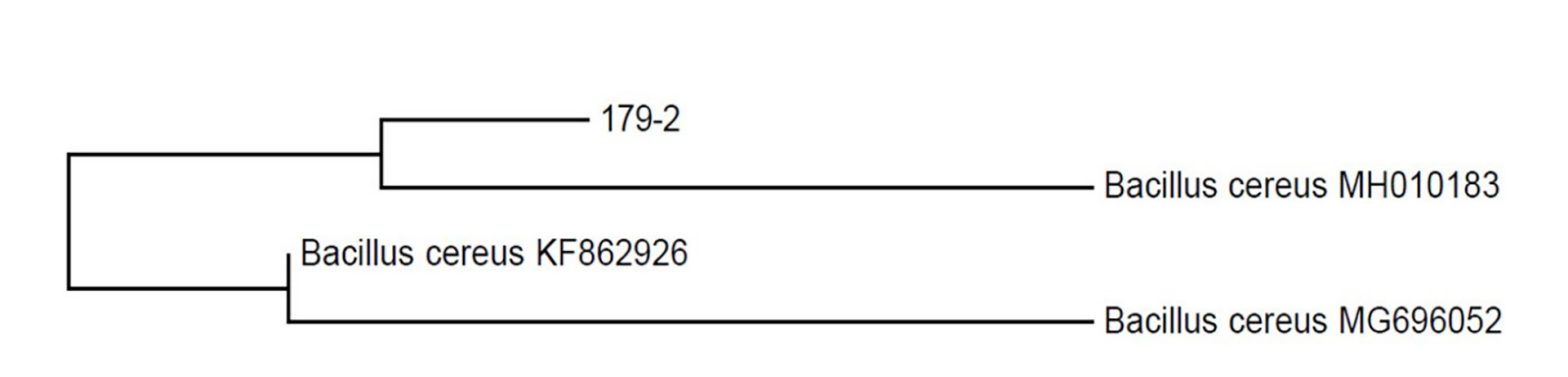
Phylogenetic tree of strain ATA179 based on the neighbor-joining method.

Isolate was identified as *Bacillus cereus* based on 16S rRNA sequence similarly (Figure 2). The partial 16S rRNA of the isolate ATA179 was deposited in GenBank (accession number MW699624). It was named *B. cereus* ATA179.

**Figure 3:**
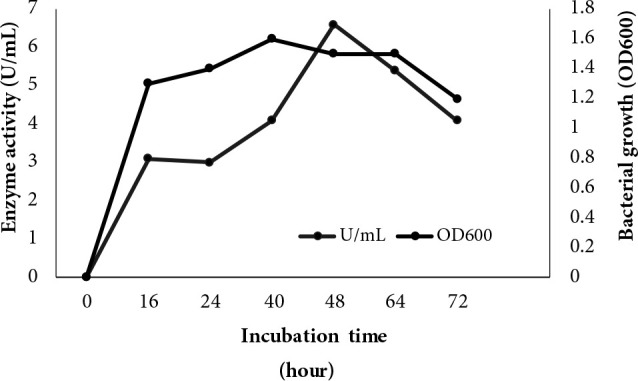
Lipase production capacity and the time-dependent changes of the reproductive values of B. cereus ATA179 in medium 3 (Kumar et al., 2005).

The maximum enzyme production of ATA179 was obtained with 6.6 U/mL at 48 h in the medium of Kumar et al. (2005) (Figure 3). The maximum biomass reached after 40 h cultivation.

### 3.2. Medium optimization

**Figure 4:**
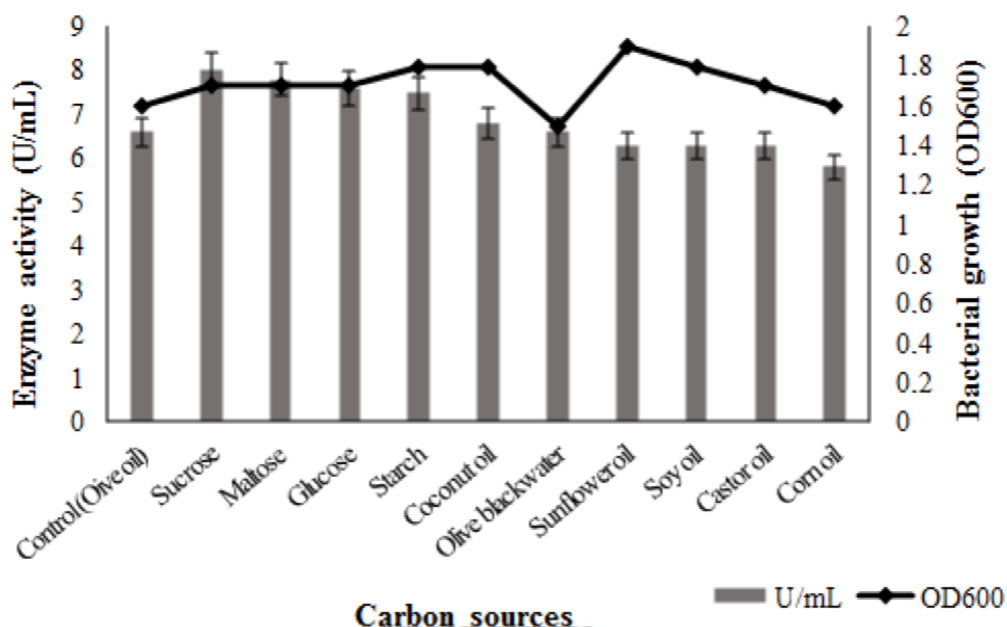
Effects of carbon sources on bacterial growth and lipase production. Carbon sources were used as 1% in Kumar et al. (2005) medium. The each flask was inoculated with 1% overnight culture (OD600 = 0.3) and incubated at 37  C for 48 h in a shaking incubator (150 rpm). Results are means of three independent determinations. Bars correspond to standard deviation.

Ten carbon sources were tried for lipase production. The best one was sucrose (8 U/mL). An increasement of 21% in lipase production was achieved compared to the control medium. The carbon source preference ranking of ATA179 in terms of enzyme production was as follows; sucrose > maltose > glucose > starch > coconut oil > olive blackwater = control (olive oil) > sunflower oil = soy oil = castor oil > corn oil (Figure 4). Maximum bacterial growth was as follows; sunflower oil> starch = soy oil = coconut oil> glucose = sucrose = maltose = castor oil> corn oil = control> olive blackwater (Figure 4).

**Figure 5:**
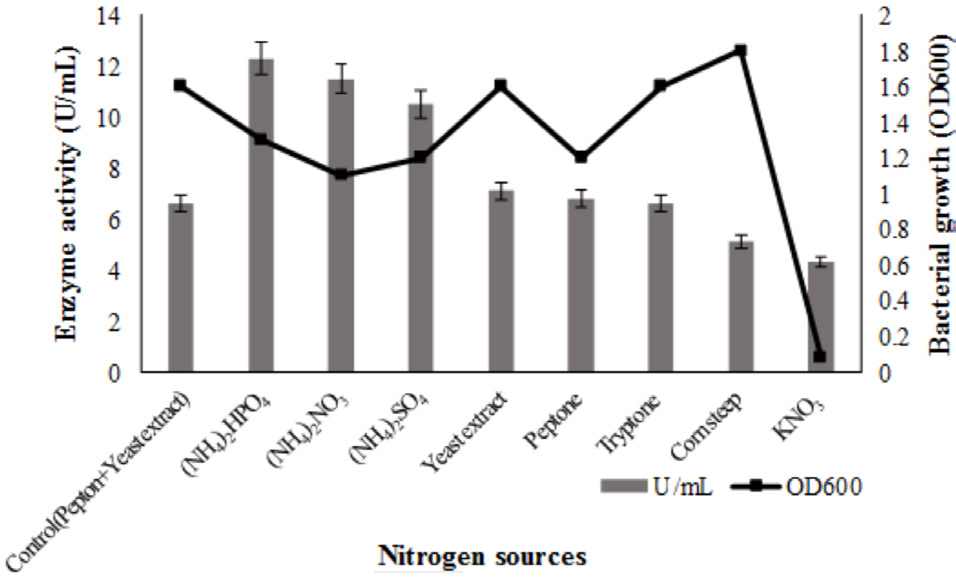
Effects of nitrogen sources on bacterial growth and lipase production. Organic and inorganic nitrogen sources were used as 1% in Kumar et al. (2005) medium. The each flask was inoculated with 1% overnight culture (OD600 = 0.3) and incubated at 37  C for 48 h in a shaking incubator (150 rpm). Results are means of three independent determinations. Bars correspond to standard deviation.

Effect of various nitrogen sources on lipase production was tested. The results showed that the best production was determined as 12.3 U/mL in the presence of (NH_4_)_2_HPO_4 _as the inorganic source. The enzyme yield was 86% compare to the control medium. In addition, enzyme yield was achieved with 74% (NH_4_)_2_NO_3_ and 59% (NH_4_)_2_SO_4_. Organic and inorganic nitrogen sources preference in terms of enzyme activity of *Bacillus cereus* ATA179 were (NH_4_)_2_HPO_4 _> (NH_4_)_2_NO_3 _> (NH_4_)_2_SO_4 _> yeast extract > peptone > tryptone = control > corn steep > KNO_3_ (Figure 4). Inorganic sources were found to be more effective than organic sources but the least enzyme activity was observed with KNO_3_. The maximum bacterial growth was as follows: corn steep > yeast extract = tryptone = control > (NH_4_)_2_HPO_4 _> peptone = (NH_4_)_2_SO_4 _> (NH_4_)_2_NO_3 _> KNO_3_ medium (Figure 5). 

**Figure 6:**
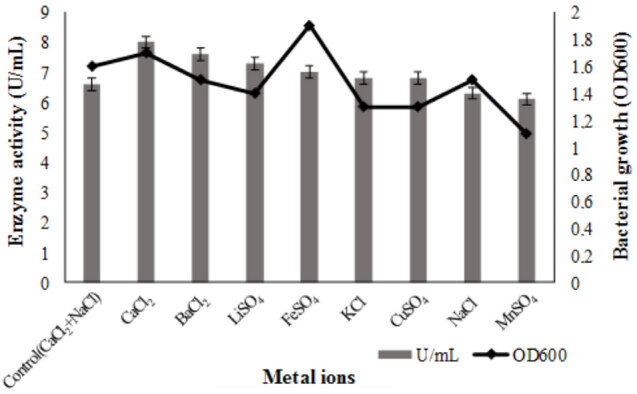
Effects of metal ions on bacterial growth and lipase production. Metal sources were used as 0.055% in Kumar et al. (2005) medium. The each flask was inoculated with 1% overnight culture (OD600 = 0.3), and incubated at 37  C for 48 h in a shaking incubator (150 rpm). Results are means of three independent determinations. Bars correspond to standard deviation.

The effect of different metal ions was assessed. CaCl_2 _(8 U/mL) was found to be the best metal source (Figure 6), and an enzyme yield of 21% was obtained. All metal ions have been found to be equally effective. When CaCl_2_ and NaCl in the control medium were taken separately, CaCI_2_ was found to be more effective alone.

**Figure 7:**
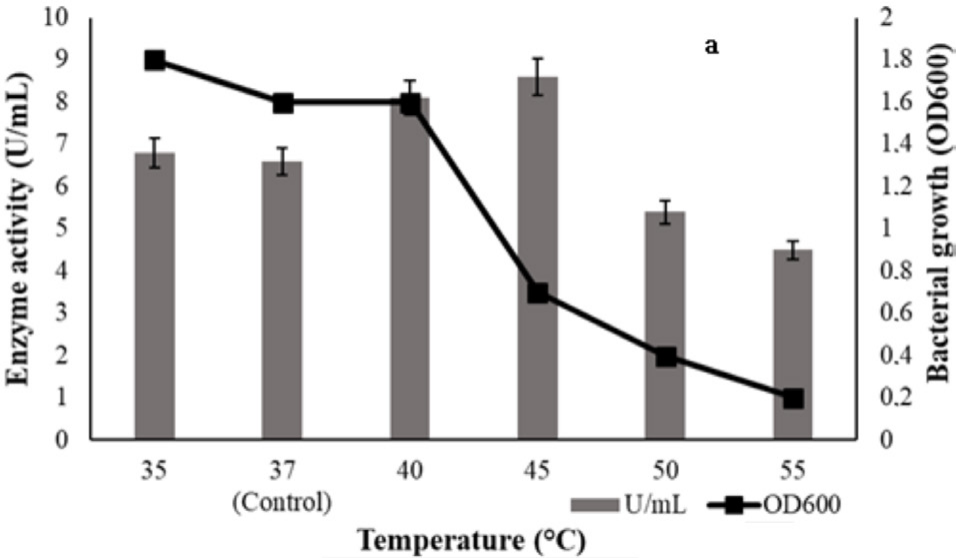

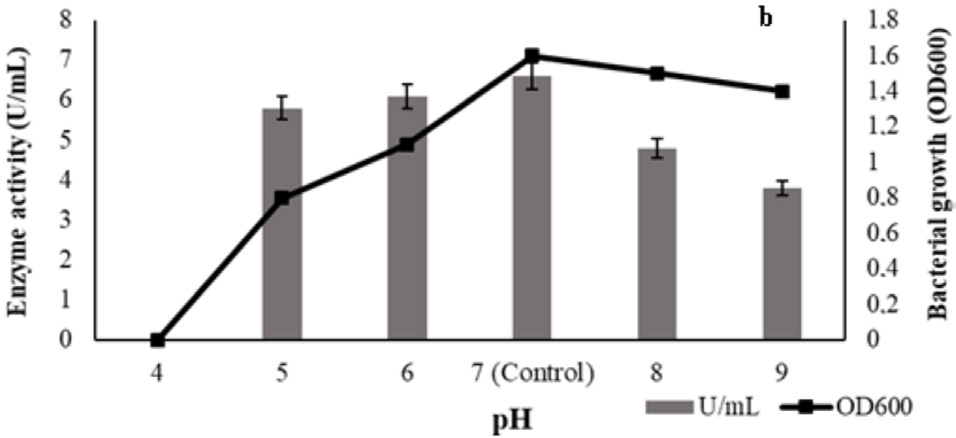

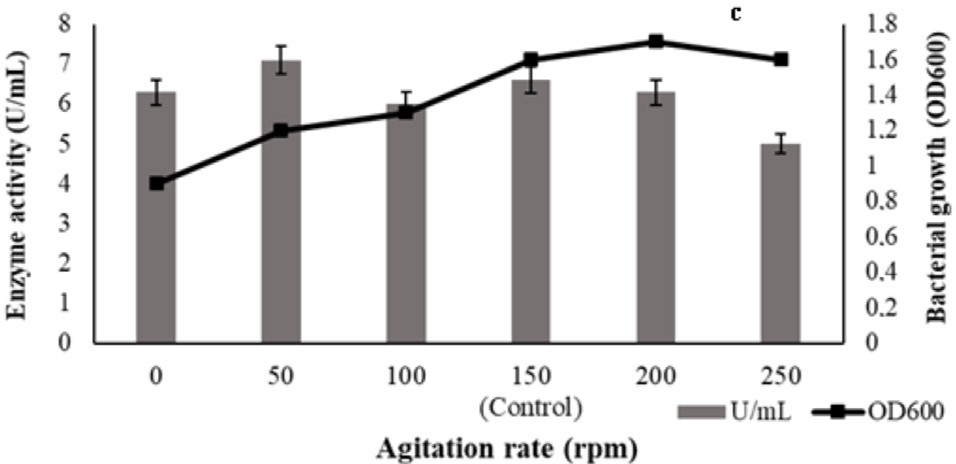

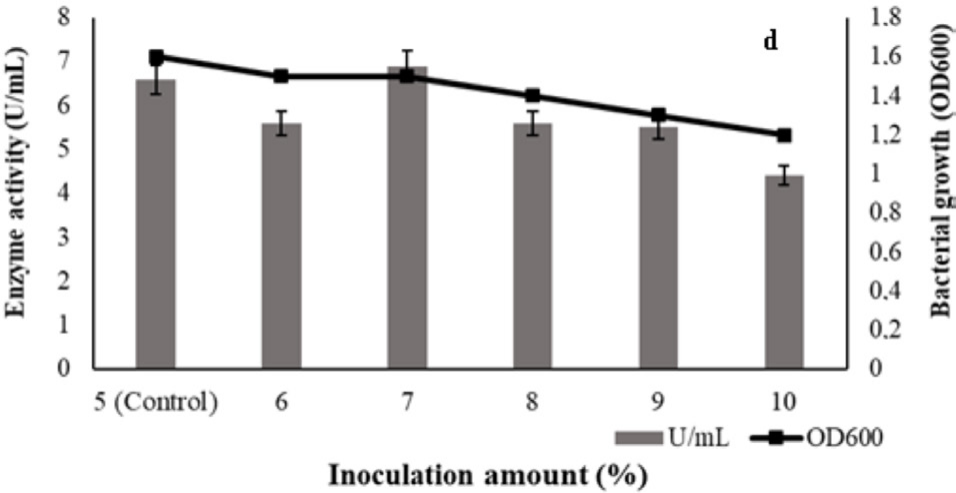

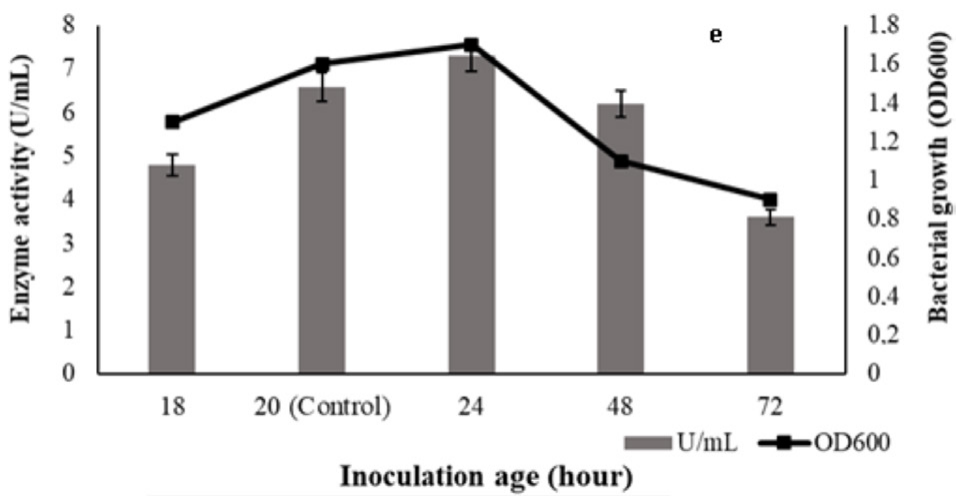
Effects of temperature (a), pH (b), agitation rate (c), inoculation amount (d), inoculation age (e) on bacterial growth and lipase production. Agitation condition was carried out at following shaking rate 0 rpm, 50 rpm, 100 rpm,150 rpm, 200 rpm, and 250 rpm. Inoculum amounts were 5%, 6%, 7%, 8% , 9%, and 10%. Inoculum ages were 18 h, 20 h, 24 h, 48 h, and 72 h. Each assay was done in Kumar et al. (2005) medium and incubated at 37  C for 48 h. Results are means of three independent determinations. Bars correspond to standard deviation.

Some physical factors (temperature, pH, agitation, inoculum amount and inoculum age) were studied for its influence on lipase production by ATA179. In the study, it was determined that the maximum enzyme production was at 45 °C (Figure 7a). However, biomass was found to be low at the same degree. Various pH values were tested and maximum enzyme production was reached at pH = 7.0 (Figure 7b). No growth was observed at pH = 4.0. While more growth was detected at high pH, decreases in enzyme production were determined. The optimum agitation rate for lipase production was 50 rpm (Figure 7c). While there was a gradual decrease in enzyme production with increasing agigation rates, an increase in growth was observed. Obtained results showed that the optimum inoculum amount for maximum lipase production was 7 %. (Figure 7d). Bacterial growth remained almost stable. In this study, the maximum enzyme production was obtained with 24 h culture as inoculum age (Figure 7e). Bacterial growth and enzyme production decreased with increasing age of inoculation. 

In this study, a new production medium was developed by optimizing nutritional and physical factors for lipase from *B. cereus* ATA179 strain. The modified medium contains 1% sucrose, 1% (NH_4_)_2_HPO_4_ and 0.055% CaCl_2_. This medium was inoculated at a rate of 7% from the culture whose inoculation age was 24 h and the production was carried out at 45 °C, pH = 7.0, 50 rpm for 48 h. The enzyme activity was determined as 10 U/mL. An enzyme yield of 51.5% was obtained compared to the control (6.6 U/mL) medium. There was also an increase in bacterial growth.

### 3.3. Partial purification and characterization 

**Figure 8:**
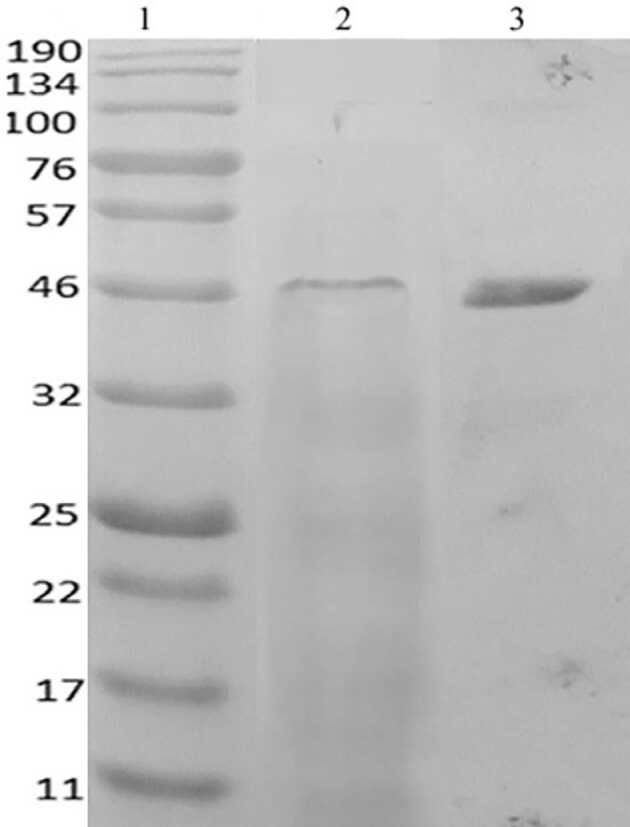
SDS-polyacrylamide gel electrophoresis of lipase. Lane 1. Protein markers (between 11 and 190 kDa), Lane 2. Crude enzyme extract, Lane 3. Partially purified enzyme.

 Partial purification of lipase was realized with ammonium sulphate precipitation (70%). After dialysis, the enzyme was partially purified by ultrafiltration. The lipase was 7.2 fold partially purified, and obtained in a 5.7% yield. Specific activity was 36.5 U/mg. The results of partial purification of lipase from *B. cereus* ATA179 are summarized in Table 1. The purity was checked by SDS-PAGE, and the molecular weight was estimated at 47 kDa (Figure 8).

**Table 1:** Summary of partial purification of lipase.

Purification steps	Total Protein (mg)	Total Lipase Activity (U)	Specific Activity (U/mg)	Yield (%)	Purificationfold
Crude extract	130	660	5.07	100	1
Amm. Sulphate Saturation (70%)	15.6	200	12.8	30	2,5
Dialysis	6.72	147	21.8	22	4.2
Ultrafiltration	1.04	38	36.5	5.7	7.2

**Figure 9:**
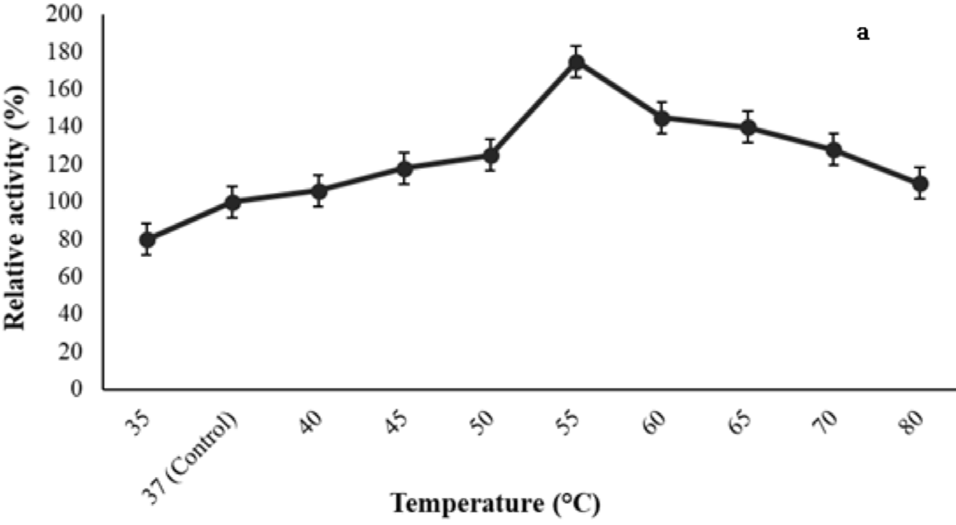

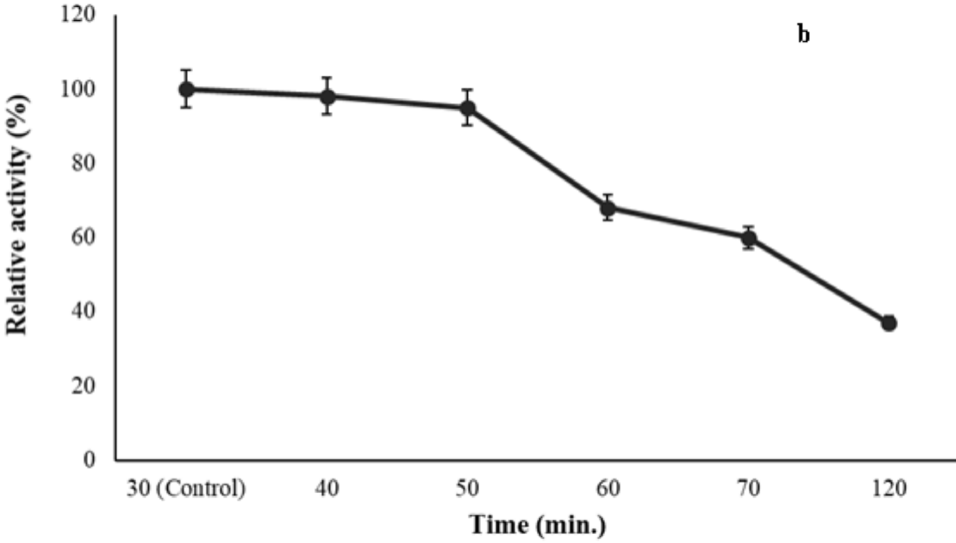

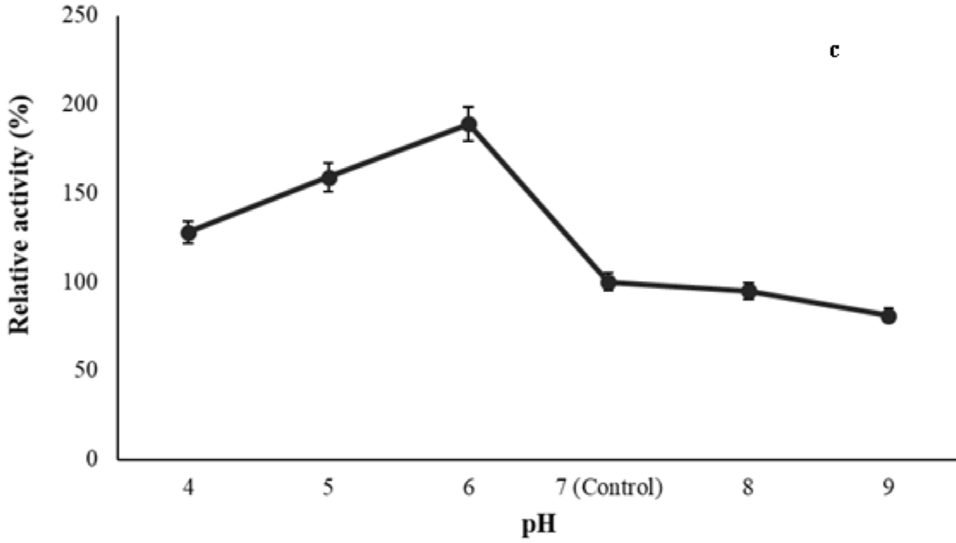

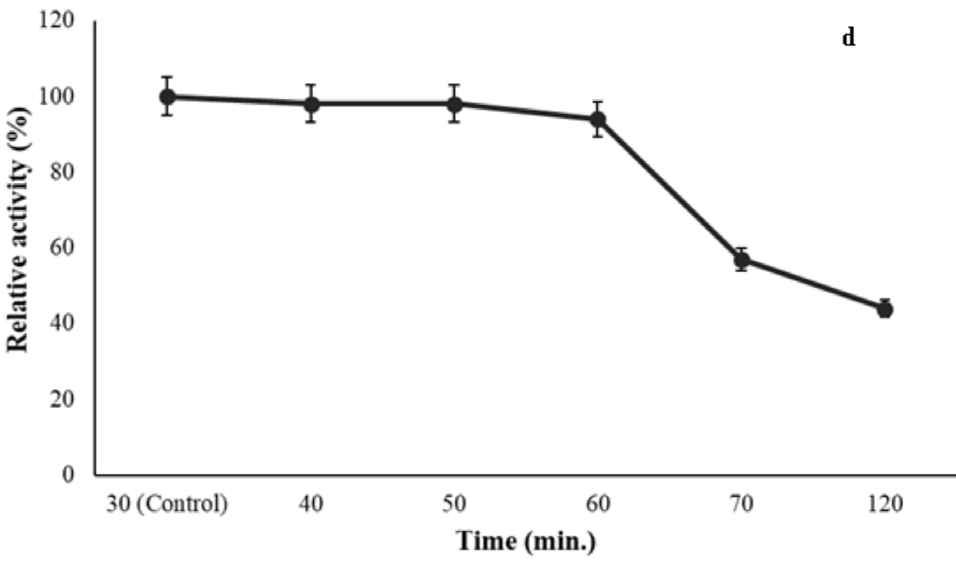
Effect of temperature on enzyme activity (a) and stability (b). For optimal temperature, the enzyme was incubated at different temperatures under the standard assay conditions. Thermostability of lipase was determined at 55  C for 2 h. pH value effect on lipase activity (c) and stability (d). For optimal pH value, enzyme was incubated in different buffer solutions at various pH values ranging under the standard assay conditions. pH value stability was determined for 2 h at pH = 6.0. The activity values were calculated as% relative activity compared with the control value (100%). Bars represent means   standard deviations for three replicates.

The optimum temperature for the lipase activity from *Bacillus cereus* ATA179 was 55 ºC (Figure 9a). Thermostability studies have shown that the activity of lipase was retained at 90% at 55 °C for 50 min (Figure 9b). Therefore, it may be a thermostable enzyme. As seen in Figure 9c, it was determined that the optimum pH value of the enzyme was 6.0. While it was determined that the enzyme has high activity in acidic side, decreases in activity were observed in alkaline side. When the pH stability was investigated, it was found that it remained active (94%) for 60 min at pH = 6.0 (Figure 9d).

**Figure 10:**
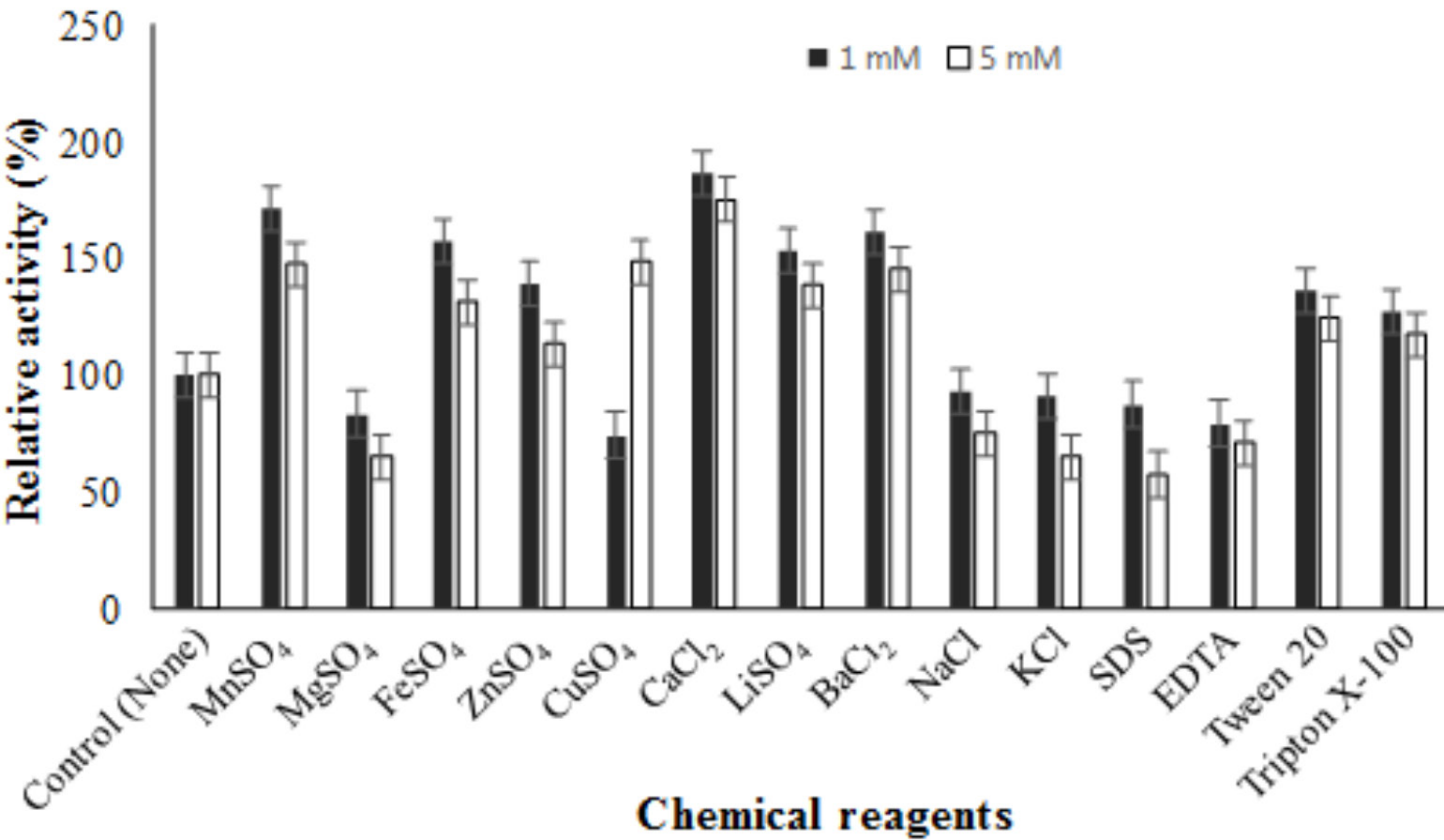
Effects of metal ions and chemical reagents on lipase activity. The activity was analysed by incubating the enzyme in the presence of various metal ions and chemical reagents (1 and 5 mM) under optimal assay conditions. The activity values were calculated as % relative activity compared with the untreated control value (100%). Bars represent means   standard deviations for three replicates.

**Figure 11:**
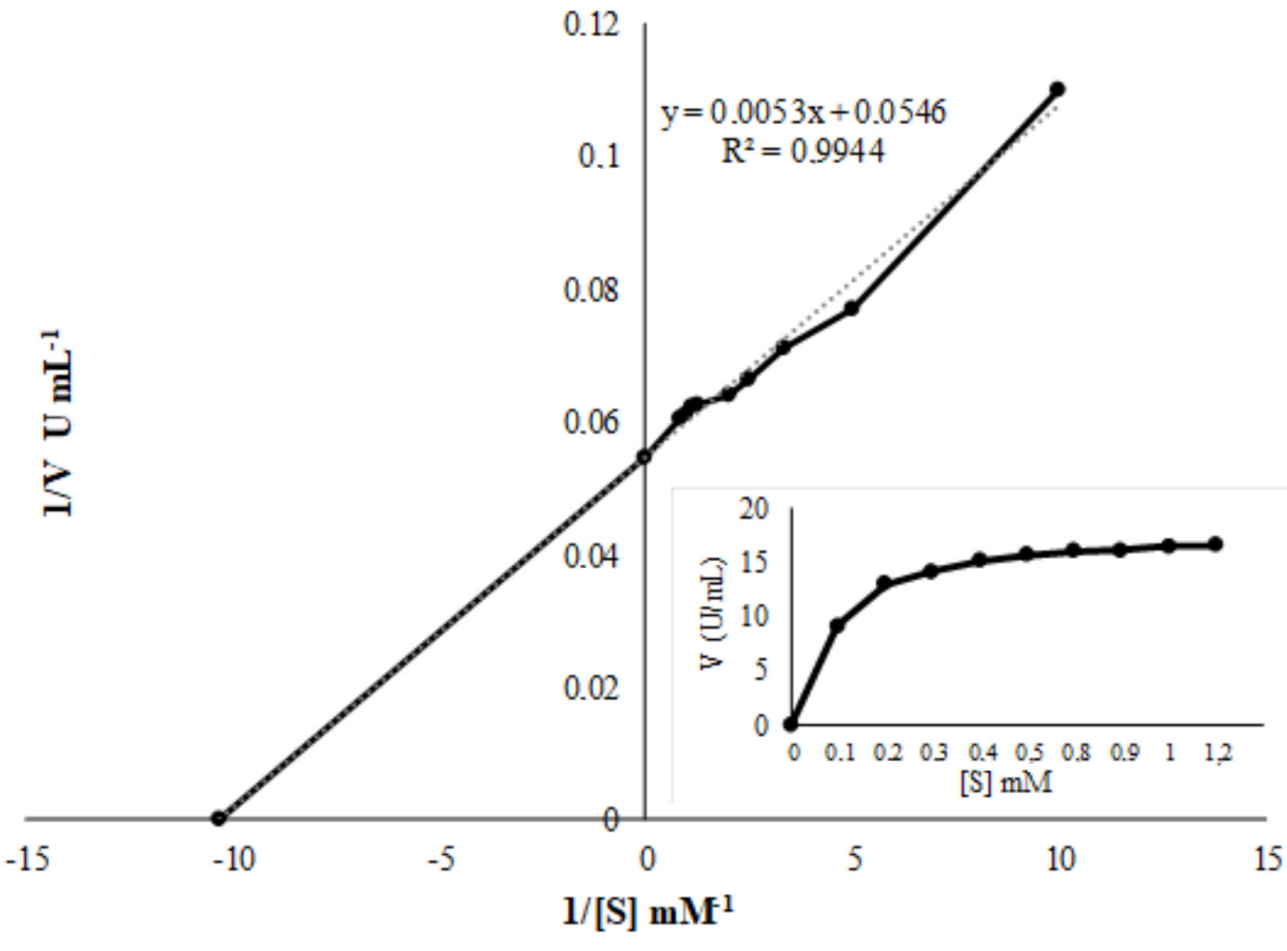
Lineweaver-Burk plot and Michaelis-Menten graph used to estimate kinetic constants of partially purified lipase.

The effects of some potential compounds on enzyme activity were studied to find out which one of them were inhibitor or stimulator. CaCl_2_, MnSO_4_ and BaCl_2_stimulated lipase activity at both 1 and 5 mM concentration. Overall, the metal ion concentration of 1 mM was found to be more effective than that of 5 mM. NaCl and KCl showed an inhibitory effect. The lipase activity was activated by Triton *×* 100 and Tween 20, inhibited by EDTA and SDS (Figure 10). The K_m_ and V_max _of lipase from *B. cereus* ATA179 were calculated as 0.11 mM and 18.2 U/mL, respectively (Figure 11).

### 3.4. Storage stability of the crude lipase 

**Figure 12:**
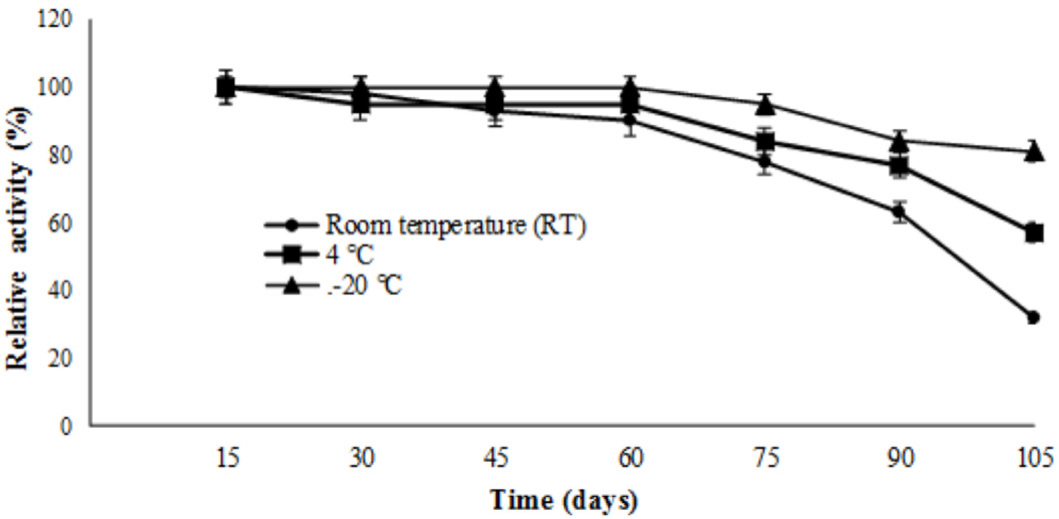
Storage stability of lipase. To determine the storage stability of enzyme, enzyme solution was stored at room temperature, 4  C and –20  C, the activity was measured at each 15 days up to 105 days under standard assay conditions. The change in lipase activities were given as relative activity (%) calculated according to the initial activities. Bars represent means   standard deviations for three replicates.

The enzyme remained more stable at –20 °C in the experiments made to determine the storage temperature. After 105 days, the enzyme retained 81% of its initial activity. The activity of the enzyme was almost preserved upto 60 days at room temperature (RT) and 4 °C (Figure 12).

### 3.5. Effects of detergent additives

The stability of the lyophilized lipase in the presence of various oxidizing agents and surfactants was checked to determine the potential of the lipase in the detergent industry. Triton X-100, EDTA, SDS and H_2_O_2_ were added at 1% concentrations, and the lipase enzyme was incubated with these detergent additives for 1 h at 100 rpm, 55 and 65 °C. According to the initial activity of the enzyme, as seen in Table 2, enzyme activity increased 22% in the medium containing 5% SDS at 65°C.

**Table 2:** Effects of 1 and 5% concentrations of detergent additives at different temperatures on stability of the lyophilized lipase enzyme.

Detergent Additives	Relative activity at 55°C (%)		Relative activity at 65°C (%)	
	1% concentration	5% concentration	1% concentration	5% concentration
SDS	100	108	113	122
Triton X-100	111	103	113	116
H2O2	113	103	108	106
EDTA	103	113	113	113

### 3.6. Effect of detergents and lipase on oil removal

**Figure 13:**
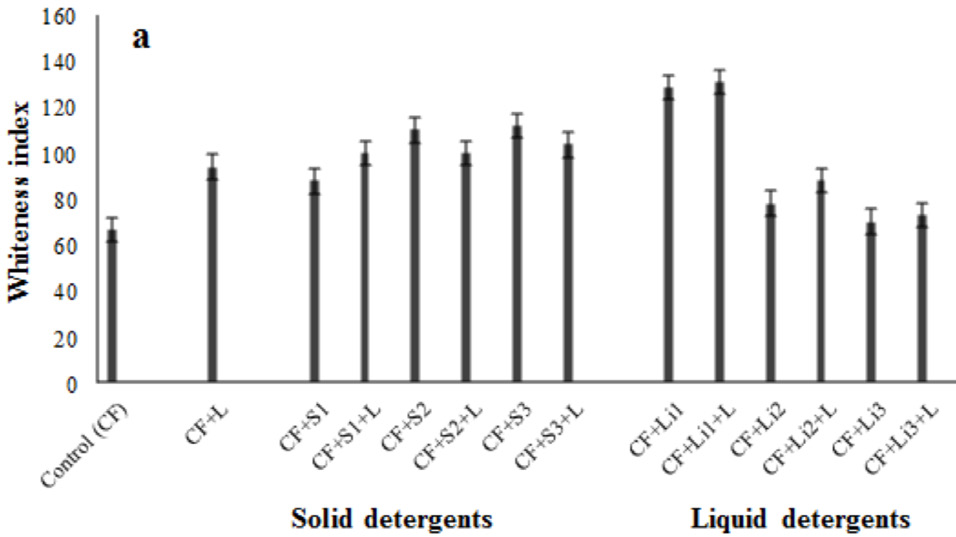

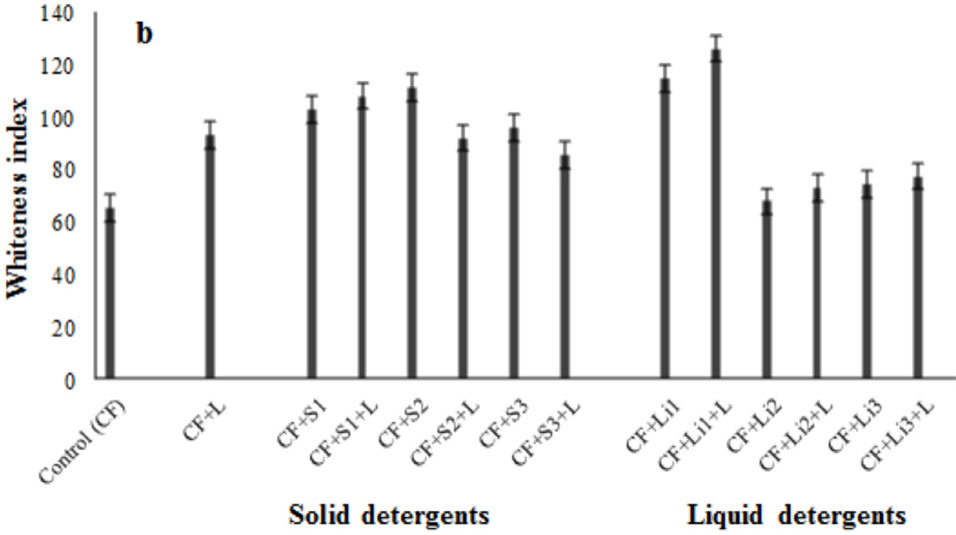
Effect of lipase alone and in the presence of solid - liquid detergents on fabrics contaminated with olive oil (a) and grease oil (b). Three different brands of liquid and solid detergents were used. The fabrics treated were incubated at 37  C for 1 h. After incubation, the fabrics were gently rubbed with a brush and rinsed with distilled water and dried. The “whiteness” indexes of the control and treated samples were determined by color measurement spectrophotometer. The bars represent means   standard deviations for three measurements. CF (contaminated fabric), L (lipase), S1, S2, S3 (solid detergent brands), L1, L2, L3 (liquid detergent brands).

The potential effect of lipase enzyme on fabrics contaminated with olive oil and grease oil was investigated. According to the results, the whiteness index of only olive oil and grease oil contaminated fabrics was obtained as 66.6 and 65.4, respectively. In the experiment where only lipase was used, the whiteness index was 94 in olive oil fabric and 92.9 in grease oil fabric (Figure 13a and 13b). As compared to the measurements made after applying lipase, 41%–42% whiteness index was detected. This has shown that the lipase enzyme is effective. In both pollutants, when lipase is combined with solid and liquid detergents, it has been observed that it has good interaction with some detergents and less effect in some. The less effective effect of enzyme combined with some detergents may be due to the chemical formulation of detergents as the additives contained in detergents may have reduced the enzyme’s activity. In this study, the best effect of lipase enzyme was obtained when used with L1 detergent on grease oil. The whiteness indices of liquid detergent (L1) in fabrics contaminated with olive oil and grease oil were determined as 128.5 (93% yield) and 114.5 (75% yield), respectively. If this detergent was used together with lipase enzyme, it was determined that the result would be 131 (97% yield) and 126 (93% yield), respectively. But in general, lipase was effective in the presence of solid detergents (Figure 13a and 13b). 

## 4. Discussions

Industrial enzymes have gained importance due to the diversity of their usage areas and their high economic value. Since each industrial application needs enzymes with specific features, the microorganisms that produce these enzymes must be isolated from nature. Among these enzymes, lipases have a wide range of applications in the pharmaceutical , food, and detergent industries (Houde et al., 2004).

In this study, lipase capacities by *Bacillus* sp. strains isolated from soil samples were screened qualitatively and lipase potential of 141 strains from 341 *Bacillus* sp. strains were determined. Among them, the *Bacillus* sp. strain with the highest lipase production was identified at the species level by 16S rRNA sequence analysis and was named *Bacillus cereus* ATA179. 

Since the promotion of lipase enzyme production depends on the nutrients in the medium where the bacteria are place, 3 different media were used and the best medium for enzyme production was determined. The new isolate showed the highest lipase production after 48 h incubation (6.6 U/mL). The highest growth was obtained at 40th h. It was determined that the maximum enzyme production was in stationary phase. In other studies with different *Bacillus* species, maximum production was obtained at different hours. It was achieved at 16 h for *B. thermocatenulatus* (Schmidt-Dannert et al., 1997), at 12 h for *Bacillus* RSJ1 (Sharma et al., 2002) , while *Bacillus* strain, *B. cereus* and *B. coagulans* had maximum lipase production at 72 h (Sarkar et al., 1998), and at 60 h for *Bacillus methylotrophicus* PS3 (Sharma et al., 2017). Chakraborty and Raj (2008) obtained maxima lipase production by *B. licheniformis* MTCC 6824 after 48 h incubation. It was reported that the highest lipase production was in the logarithmic phase, the end of the logarithmic phase, or the stationary phase.

The content of the culture medium and fermentation conditions are important factors affecting enzyme production. The culture media has a striking effect as it stimulates enzyme production. Since lipases are inducible enzyme, they are significantly affected by lipid, carbon, and nitrogen sources. In this study, nutritional factors were assessed, the best carbon source was sucrose and enzyme production increased by 21% compared to basal medium. While enzyme production increased in the presence of maltose, glucose and starch, it was not stimulated in the presence of lipid sources. Among nitrogen sources, the best was inorganic nitrogen source (NH_4_)_2_HPO_4_ with a yield of 86%. Enzyme yield was achieved by 74% with (NH_4_)_2_NO_3_, 59% with (NH_4_)_2_SO_4_ and 7.5% with the organic nitrogen source yeast extract. Among the nitrogen sources, inorganic sources other than KNO_3_ were more effective than organic sources. This can be due to the positive interaction of ammonium salts with other components in the medium. Some metal ions are an important factor affecting enzyme production because they act as stimulator. In the study, CaCl_2_ was more effective, followed by BaCl_2_ and LiSO4 to determine the effect of metal ions. MnSO_4 _andNaCl ions has the lowest ability of enzyme production. CaCl_2_ and NaCl, which are found together in the basal medium, has been tested separately, CaCl_2 _showed a 21% increase in enzyme production when used alone. The presence of more than one metal ions in the medium may have a unfavorable action on production. 

Physical factors are as effective as nutritional factors in enzyme production. The lipase production by** ATA179 was optimized in terms of, temperature, pH, inoculum amount, agitation and inoculum age. The optimum temperature value was detected as 45 °C. The enzyme production at 45 and 40 °C increased by 30% and 22% compared to the control 37 °C, respectively. The optimum pH value was found to be 7.0. The enzyme production declined in below and above of pH 7.0. Agitation provides nutrients and oxygen to spread homogeneously in the medium. The best agitation was 50 rpm and, a 7.5% efficiency increase was determined. The inoculum amount was an important factor in an experimental design to obtain high enzymatic activity was an important factor in an experimental design to obtain high enzymatic activity (Kammoun et al., 2008). In this study, the cultivation was carried out at different inoculum amount (5%–10%). The optimum inoculum amount was found to be 7%, as compared to 5% inoculum used initially and showed a 4.5% increase in production. An optimum inoculum amount will be suitable for bacterial growth in an medium with sufficient oxygen and nutrients. It was found that the increase of the amount of inoculation was not effective on enzyme production. Influence of inoculum age on lipase production was investigated. The inoculation age at which the maximum enzyme production was obtained was determined as 24 h. An increase of 10.6% has been achieved compared to the control. The use of old cultures as inoculum caused a decrease in production which may be due to the old cultures late adapting to the medium. In this study, a new modified medium was created by combining the best nutritional and physical parameters for the production of high amounts of lipase from the new ATA179 isolate. In this new medium 51.5% lipase production was achieved compared to basal medium.

The age of inoculum is highly variable depending on the process, cultivation conditions, medium composition and the microorganism, among other factors. Moreover, the inoculum amount was an important factor in an experimental design to obtain high enzymatic activity (Veerapagu et al., 2014).

Various researchers have investigated the effects of nutritional and physical factors on lipase production and reported different or similar results. Lipase production was stimulated with 2% starch and olive oil. Maximum enzyme production conditions were found to be 37 °C, neutral pH and 24 h incubation (Alkan et al., 2007). Kumar et al. (2012) reported that the best nutritional and physical factors for lipase production from *Bacillus* MPTK 912 were glucose, peptone, Fe^2+^ and Mg^2+^, pH 8.0 and temperature 35 °C. Sirisha et al. (2010) stated that peptone was the best nitrogen source for lipase production. Similar to our result, Dong et al. (1999) reported that inorganic nitrogen sources (NH_4_Cl and (NH_4_)H_2_PO_4_) were more efficient. Bacha et al. (2016) noted that xylose and yeast extract were the best sources of carbon and nitrogen for lipase production from *S. aureus* . Many researchers have reported that glucose has different effects. While glucose stimulates lipase production in *B. licheniformis* H1, *Bacillus sp* . GK 8 and SB-3 (Bradoo et al., 1999; Dosanjh and Kaur, 2002), *B. alcalophilus* B-M20 (Ghanem et al., 2000) *, B. megaterium* AKG-1 (Sekhon et al., 2006 **),** *Bacillus.* sp. L2 (Shariff et al., 2007) and *B. subtilis* (Mormeneo et al., 2008) strains have been found to inhibit lipase production. On the other hand, lipase production from *Bacillus* strains was induced by fats but its expression was stimulated by sugars and sugar alcohols, especially galactose, lactose, glycerol, and mannitol (Gupta et al., 2004). In our study, it was found that enzyme production was not high in the presence of fats. Oil hydrolysis products may have had a negative effect on lipase production. However, olive oil and yeast extract was found to be most effective on lipase production from *Aspergillus niger* . Optimum physical parameters were 24 °C, pH = 7.0, 200 rpm and 72 h (El-Batal et al., 2016).

The best lipase production from *Bacillus thuringiensis* (TS11BP) was at pH = 8.0, 45 °C, 96 h with 14% inoculum amount. Dextrose as carbon source and beef extract as nitrogen source were reported (Duza and Mastan, 2014). Pallavi et al. (2014) stated that starch and peptone were the best sources for *Bacillus subtilis* Y-IVI strain. Mazhar et al. (2017) reported that the maximum lipase production from *B. subtilis* PCSIR-39 was achieved in the presence of sucrose. They found that peptone was the best nitrogen source. They reported that Ca^2 +^ and Mg^2^^+^ had good stimulating effect on enzyme production, 45 °C was the best temperature and optimal pH value was 7.0, 5% of inoculum. Niyonzima et al. (2013) optimized the lipase production environment for *Bacillus flexus* XJU-1. They achieved maximum lipase production in 36 h, at 37 °C, pH = 11.0, 2% inoculum amount, when they used refined yeast extract as the best nitrogen source and cottonseed oil as the best carbon source. While *Bacillus* L2 lipase production was totally inhibited by Mg^2 +^ ions, the addition of Ca^2 +^ and Fe^3^^+ ^resulted in high lipase production (Shariff et al. 2007). *B. alcalophilus* B-M20 can tolerate salinity up to 7.5 %, but high NaCl and KCl concentrations inhibit lipase production (Ghanem et al., 2000).

The optimal temperature, pH value ,and agitation rate of *Staphylococcus hominis* MTCC 8980 were found to be 33.1 °C, 7.9, and 178.4 rpm, respectively (Behera et al., 2019). While maximum lipase production from the *Bacillus* sp. strains is achieved at pH = 6.0 and 37 °C (Bharathi, 2019), Larbidaouadi et al. (2015) have achieved it at pH = 8.0 and 40 ^o^C (1.5 U/mL). Iftikhar et al. (2003) reported the amount of inoculum as 3.0%. Sekhon et al. (2006) stated the range of 6.5-8.0 as the enzyme production pH value. In *Bacillus tequilensis* NRRL B41771 (Bonala and Mangamoori, 2012), 1% inoculum amount has been reported for optimal lipase production. In contrast, *Bacillus pumilus* has a 10% higher inoculation amount for maximum lipase production (Heravi et al., 2008). Kumar et al. (2005) have prepared a new modified medium for *Bacillus coagulans* BTS-3. In the combination of peptone and yeast extract as nitrogen sources and mustard oil as carbon source at pH = 8.5, 55 °C and 48 h, enzyme activity was determined as 1.16 U/mL. Abbas et al. (2017) reported that maximum lipase production (12.81 U/mL) from *Bacillus subtilis* PCSIR NL-38 was obtained with NH_4_NO_3, _glucose, and FeSO_4_.7H_2_O at pH = 7.0, 40 °C, 7% inoculum amount and 48 h of incubation. In the study with *Bacillus subtilis* , lipase production reached a maximum at 30 °C with 84 h of fermentation (4.72 U/mL). They obtained a new modified medium, and lipase activity was determined as 4.96 U/mL in the presence of 5% inoculum amount, 0.5% yeast extract, 0.25% olive oil and 10 mM Ca^2 +^ (Suci et al., 2018). 

As seen in results of studies, the fact that the differences in lipase production yields with different microorganisms can be result of bacterial strain characteristics as well as the culture medium cultivation and composition conditions. In addition, different results can be obtained in measuring the lipolytic activity due to experimental conditions. In studies that investigate the effects of physical and nutritional factors on lipase production, different results show that the metabolic pathways used by microorganisms are different.

In this study conducted to determine the potential of lipase use in the detergent industry, it was found that lyophilized lipase preserved its stability with oxidizing agents and various surfactants. On the other hand, in determining the effect of lipase enzyme on fabrics contaminated with olive oil and grease oil, the whiteness index measurements showed positive results on the removel of contamination. Our results suggest that the lipase from new isolate ATA179 may have potential in the detergent industry.

## Compliance with ethical standards

Authors declare that they have no conflict of interest and procedures including human and/or animal subjects.

## References

[ref1] (2017). Lipase Production from Bacillus subtilis using various Agricultural waste. International Journal of Advanced Engineering, Management and Science.

[ref2] (2004). Enzymes in Industry-Production and Applications.

[ref3] (2007). Production of lipase by a newly isolated Bacillus coagulans under solid-state fermentation using melon wastes. Applied Biochemistry and Biotechnology.

[ref4] (1990). Basic local alignment search tool. Journal of Molecular Biology.

[ref5] (2007). Optimization of process parameters for the production of lipase in submerged fermentation by Yarrowia lipolytica NCIM 3589. Research Journal of Microbiology.

[ref6] (2010). Seed lipases: sources, applications and properties-a review. Brazilian Journal of Chemical Engineering.

[ref7] (2016). An organic solvent-stable lipase from a newly isolated Staphylococcus aureus ALA1 strain with potential for use as an industrial biocatalyst. Biotechnology and Applied Biochemistry.

[ref8] (2019). Optimization of physical parameters for enhanced production of lipase from Staphylococcus hominis using response surface methodology. Environmental Science and Pollution Research.

[ref9] (2019). Optimization and Production of lipase enzyme from bacterial strains isolated from petrol spilled soil. Journal of King Saud University-Science.

[ref10] (2016). Characterization of a hyperthermostable alkaline lipase from Bacillus sonorensis 4R. Enzyme Research.

[ref11] (1999). Two acidothermotolerant lipases from new variants of Bacillus spp. World Journal of Microbiology and Biotechnology.

[ref12] (2012). Production and optimization of lipase from Bacillus tequilensis NRRL B-41771. International Journal of Biotechnology Applications.

[ref13] (2008). An extracellular alkaline metallolipase from Bacillus licheniformis MTCC 6824: Purification and biochemical characterization. Food Chemistry.

[ref14] (2020). Microbial lipases and their industrial applications: a comprehensive review. Microbial Cell Factories.

[ref15] (2014). Screening of phytate hydrolysis Bacillus sp. isolated from soil and optimization of the certain nutritional and physical parameters on the production of phytase. Turkish Journal of Biochemistry.

[ref16] (2020). Investigation of effects of protease enzyme produced by Bacillus subtilis 168 E6-5 and commercial enzyme on physical properties of woolen fabric. The Journal of The Textile Institute.

[ref17] (1999). Purification and characterization of a Pseudomonas sp. lipase and its properties in non-aqueous media. Biotechnology and Applied Biochemistry.

[ref18] (2002). Biochemical analysis of a native and proteolytic fragment of a high-molecular-weight thermostable lipase from a Mesophilic Bacillus sp. Protein Expression and Purification.

[ref19] (2014). Optimization of lipase Production from Bacillus thuringiensis (TS11BP), Achromobacter xylosoxidans J2 (TS2MCN) –Isolated from soil sediments near oilseed farm. Journal of Pharmaceutical and Biological Sciences.

[ref20] (1901). Über enzyme bei bakterien und schimmelpilzen. Centralblatt für Bakteriologie.

[ref21] (2016). Effect of environmental and nutritional parameters on the extracellular lipase production by Aspergillus niger. International Letters of Natural Sciences.

[ref22] (2012). Correlation between agar plate screening and solid-state fermentation for the prediction of cellulase production by Trichoderma strains. Enzyme Research.

[ref23] (2000). An alkalophilic thermostable lipase produced by a new isolate of Bacillus alcalophilus. World Journal of Microbiology and Biotechnology.

[ref24] (2004). A glycerol-inducible thermostable lipase from Bacillus sp. Canadian Journal of Microbiology.

[ref25] (2008). Isolation and identification of a lipase producing Bacillus sp. Pakistan Journal of Biological Sciences.

[ref26] (2004). Lipases and their industrial applications: an overview. Applied Biochemistry and Biotechnology.

[ref27] (2003). Optimization of cultural conditions for the production of lipase by submerged culture of Rhizopus oligosporus TUV-31. Pakistan Journal of Botany.

[ref28] (2008). Application of a statistical design to the optimization of parameters and culture medium for alpha-amylase production by Aspergillus oryzae CBS 819.72 grown on gruel (wheat grinding by-product). Bioresource Technology.

[ref29] (2005). Production, purification and characterization of lipase from thermophilic and alkaliphilic Bacillus coagulans BTS-3. Protein Expression and Purification.

[ref30] (2012). Production, optimization and purification of lipase from Bacillus sp. MPTK 912 isolated from oil mill effluent. Advances in Applied Science Research.

[ref31] (1970). Cleavage of structural proteins during the assembly of the head of bacteriophage T4. Nature.

[ref32] (2015). Screening selection identification production and optimization of bacterial lipase isolated from industrial rejection of gas station. International Journal of Biotechnology and Allied Fields.

[ref33] (1951). Protein measurement with the folin phenol reagent. Journal of Biological Chemistry.

[ref34] (2017). Optimized production of lipase from Bacillus subtilis PCSIRNL-39. African Journal of Biotechnology.

[ref35] (2008). Efficient secretion of Bacillus subtilis lipase A in Saccharomyces cerevisiae by translational fusion to the Pir4 cell wall protein. Applied Microbiology and Biotechnology.

[ref36] (2013). Optimization of fermentation culture conditions for alkaline lipase production by Bacillus flexus XJU-1. Current Trends in Biotechnology and Pharmacy.

[ref37] (2014). Optimization of cultural parameters for lipase production by Bacillus subtilis Y-IVI. International Journal of Current Microbiology and Applied Sciences.

[ref38] (2002). Lipases for Biotechnology. Current Opinion in Biotechnology.

[ref39] (1987). The neighbor-joining method: a new method for reconstructing phylogenetic trees. Molecular Biology and Evolution.

[ref40] (1998). Production and optimization of microbial lipase. Bioprocess Engineering.

[ref41] (2018). Recent advances on sources and industrial applications of lipase. Biotechnology Progress.

[ref42] (1997). Two novel lipases from the thermophile Bacillus thermocatenulatus: Screening, purification, cloning, overexpression and properties. Methods in Enzymology.

[ref43] (2006). Production of extracellular lipase by Bacillus megaterium AKG-1 in submerged fermentation. Indian Journal of Biotechnology.

[ref44] (2007). Production of L2 lipase by Bacillus sp. strain L2: Nutritional and physical factors. Journal of Basic Microbiology.

[ref45] (2002). Production of extracellular alkaline lipase from a Bacillus sp. RSJ1 and its application in ester hydrolysis. Indian Journal of Microbiology.

[ref46] (2017). Purification and characterization of lipase by Bacillus methylotrophicus PS3 under submerged fermentation and its application in detergents industry. Journal of Genetic Engineering and Biotechnology.

[ref47] (2010). Isolation and Optimization of Lipase Producing Bacteria from Oil Contaminated Soils. Advances in Biological Research.

[ref48] (2018). Lipase production from Bacillus subtilis with submerged fermentation using waste cooking oil. Environmental Earth Sciences.

[ref49] (1991). Purification and Characterization of a novel thermostable lipase from Bacillus sp. Journal of Biochemistry.

[ref50] (1994). Improving the sensitivity of progressive multiple sequence alignment through sequence weighting, position-specific gap penalties and weight matrix choice. Nucleic Acids Researches.

[ref51] (2013). The Effect of Growth Parameters on the Antibıotic Activity and Sporulation in Bacillus spp. Isolated from Soil. Journal of Microbiology, Biotechnology and Food Sciences.

[ref52] (2004). Enzymes in Industry: Production and Applications.

[ref53] (2014). Isolation and identification of a novel lipase producing bacteria from oil spilled soil. International Journal of Innovative Research in Science, Engineering and Technology.

